# TRAAP2 - TRAnexamic Acid for Preventing postpartum hemorrhage after cesarean delivery: a multicenter randomized, doubleblind, placebo- controlled trial – a study protocol

**DOI:** 10.1186/s12884-019-2718-4

**Published:** 2020-01-31

**Authors:** Loïc Sentilhes, Valérie Daniel, Catherine Deneux-Tharaux, On behalf of the TRAAP2 Study Group and the Groupe de Recherche en Obstétrique et Gynécologie (GROG), Clémence Houssin, Hugo Madar, Aurélien Mattuizzi, Benjamin Merlot, Astrid Darsonval, Bellabes Ghezzoul, Antoine Bénard, Jérôme Galet, Aurore Georget, Sophie Regueme, Caroline Roussillon, Aurélie Darmaillacq, Olivier Delorme, Laure Estève, Fatima-Zahra Makhoukhi, Benoit Elleboode, Guillaume Legendre, Didier Riethmuller, Denis Gallot, Raoul Desbrière, Florent Fuchs, Olivier Morel, Emilie Gauchotte, Norbert Winer, Vincent Letouzey, François Goffinet, Camille Le Ray, Patrick Rozenberg, Maëla Le Lous, Eric Verspyck, Céline Chauleur, Nicolas Sananes, Fanny De Marcillac, Olivier Parant, Franck Perrotin, Gilles Kayem, Elie Azria, Marie-Victoire Senat, Laurent Salomon, Laurence Bussières, Laurence Lecomte, Thomas Schmitz, Florence Bretelle, Delphine Vardon, Bassam Haddad, Caroline Bohec, Damien Subtil, Christophe Vayssiere, Norbert Winer

**Affiliations:** 1 Department of Obstetrics and Gynecology, Bordeaux University Hospital, Place Amélie Raba Léon, 33076 Bordeaux, France; 2 Production Pharmaceutique pour la Recherche Institutionnelle du Grand Ouest (PPRIGO), Brest, France; 3 Department of pharmacy, Angers University Hospital, Angers, France; 4 INSERM U1153, Obstetrical, Perinatal and Pediatric Epidemiology Research Team, Center for Epidemiology and Statistics, Sorbonne Paris Cité, DHU Risks in Pregnancy, Paris Descartes University, Paris, France

**Keywords:** Postpartum hemorrhage, Treatment, Prevention, Tranexamic acid, Cesarean and vaginal deliveries, Thrombosis, Randomized trial

## Abstract

**Background:**

An antifibrinolytic agent that blocks lysine-binding sites on plasminogen molecules, tranexamic acid reduces bleeding-related mortality in women with postpartum hemorrhage (PPH), especially administered fairly soon after delivery. According to the randomized controlled trials thus far reported for PPH prevention after cesarean deliveries (*n* = 16), women who received tranexamic acid had significantly less postpartum blood loss and no increase in severe adverse effects. These were, however, primarily small single-center studies that had fundamental methodological flaws. Multicenter randomized controlled trials with adequate power are necessary to demonstrate its value persuasively before tranexamic acid goes into widespread use for the prevention of PPH after cesarean deliveries.

**Methods/design:**

This study will be a multicenter, double-blind, randomized controlled trial with two parallel groups including 4524 women with cesarean deliveries before or during labor, at a term ≥34 weeks, modeled on our previous study of tranexamic acid administered after vaginal deliveries. Treatment (either tranexamic acid 1 g or placebo) will be administered intravenously just after birth. All women will also receive a prophylactic uterotonic agent. The primary outcome will be the incidence of PPH, defined by a calculated estimated blood loss > 1000 mL or a red blood cell transfusion before day 2 postpartum. This study will have 80% power to show a 20% reduction in the incidence of PPH, from 15.0 to 12.0%.

**Discussion:**

As an, inexpensive, easy to administer drug that can be add to the routine management of cesarean births in delivery rooms, tranexamic acid is a promising candidate for preventing PPH after these births. This large, adequately powered, multicenter randomized placebo-controlled trial seeks to determine if the benefits of the routine prophylactic use of tranexamic acid after cesarean delivery significantly outweigh its risks.

**Trial registration:**

ClinicalTrials.gov NCT03431805 (February 12, 2018).

## Background

### Rationale

#### Prevention of postpartum hemorrhage

The standard definition of a primary postpartum hemorrhage (PPH) is a blood loss of 500 mL or more in the first 24 h after childbirth, regardless of mode of delivery. It causes around 25% of all maternal deaths worldwide [1–3]. Some historical studies have reported that mean blood loss is significantly higher after cesarean than vaginal deliveries [2]. A prospective observational study in France reached a similar conclusion [4]: blood loss > 500 mL occurred in 46.8% of cesarean deliveries and > 1000 mL in 11.6%; the 95th percentile of the volume of blood loss distribution was 1300 mL [4]. Because the authors estimated blood loss mainly by the volume in the suction container from the placental delivery, these data may underestimate the true blood loss. Nonetheless, these estimates of blood loss during cesareans [4] are consistent with those reported by Sheehan et al. in their double-blind randomized controlled trial (RCT) comparing two regimens of prophylactic oxytocin at elective cesarean deliveries (*n* = 2069 in total) [5]. A calculated estimated blood loss ≥1000 mL [calculated estimated blood loss = estimated blood volume × (preoperative hematocrit – postoperative hematocrit/preoperative hematocrit (where estimated blood volume = booking weight (kg) × 85)] defined the PPH incidence that was the primary outcome in their trial. They chose this validated calculation as a quantitative objective measure to estimate blood loss [6, 7] because it is widely accepted that clinicians underestimate this loss [8, 9] and that gravimetric methods include liquor in addition to blood, which limits their accuracy, especially for cesareans [10]. They reported a calculated blood loss ≥1000 mL in around 16% in both groups of women with an elective cesarean [5]. This result is consistent with that of the French prospective study mentioned above, which estimated blood loss clinically [4].

Today, of the components of the active management of the third stage of labor, a package intended to reduce PPH, only one — the administration of a uterotonic agent — has been demonstrated to reduce the incidence of PPH by 50% [2, 3, 11].

#### Tranexamic acid

Tranexamic acid, as an antifibrinolytic agent, blocks lysine-binding sites on plasminogen molecules [12]. It reduces bleeding in elective surgery [13, 14] and mortality in trauma patients [15] and does so without any rise in the number of vascular occlusive events [13–15]. In gynecology, tranexamic acid reduces menstrual blood loss in women with menorrhagia compared with control agents or placebo [16].

In obstetrics, tranexamic acid reduces bleeding-related mortality in women with PPH, especially when administered fairly soon after delivery [17]. The WOMAN trial enrolled 20,060 women with PPH, randomly assigned them to receive tranexamic acid (*n* = 10,051) or placebo (*n* = 10,009), and included 10,036 and 9985, respectively, in the analysis [17]. Significantly fewer women treated with tranexamic acid (1.5% vs 1.9%, risk ratio (RR) 0.81, 95% confidence interval (CI) 0.65–1.00; *P* = 0.045) died due to bleeding, especially those treated within 3 h of delivery (1.2 and 1.7%, respectively, RR 0.69, 95% CI 0.52–0.91; *P* = 0.008). The groups did not differ significantly for adverse (including thromboembolic) events [17]. This team also conducted an individual data meta-analysis that showed a 10% decrease in the survival benefit of tranexamic acid associated with every 15 min delay in treatment administration during the first 3 h; after 3 h, the benefit vanished [18].

##### For prevention of postpartum hemorrhage

We searched Medline through January 1, 2019, and found 20 RCTs aimed at evaluating the preventive impact on PPH of tranexamic acid administered after delivery in addition to prophylactic uterotonics [19–38]. Only four enrolled women with vaginal deliveries [19–22], the sixteen others including women with cesarean delivery [23–38]. Except for the French Tranexamic Acid for Preventing Postpartum Hemorrhage Following a Vaginal Delivery (TRAAP) trial, which we coordinated and on which this protocol is modeled [22, 39], these RCTs took place in countries with low or middle resources (China, India, Iran, Turkey, Pakistan, and Egypt), had major methodological flaws, and their results could have been affected by their potential for selection, performance, and detection biases. Their findings must therefore be interpreted cautiously, as stressed by several authors [12, 39–45]. The TRAAP trial enrolled 4000 women at standard risk for PPH with a planned vaginal delivery of a singleton live fetus at 35 weeks or more of gestation [22]. The primary outcome — blood loss of at least 500 mL — did not differ significantly between women randomized in the tranexamic acid group and those in the placebo group (RR, 0.83; 95% CI, 0.68 to 1.01; *P* = 0.07).

Sixteen RCTs have thus assessed the effect of tranexamic acid in preventing PPH in women who had elective cesareans; they reported a significantly reduced blood loss in those randomized to receive tranexamic acid, with no effect on the cardinal vital signs or on thrombosis [23–38]. Nevertheless, because of their notable methodologic concerns related to blinding, outcome assessment, and attrition bias, their findings must be considered inconclusive. Furthermore, the external validity of the results observed in middle-income countries appears uncertain, and they lacked adequate power to assess severe adverse events [12, 39–45]. The interpretation of their results thus requires particular caution.

Finally, it is important to emphasize that none of these RCTs included cesarean deliveries during labor [12], when moderate and severe blood loss are both significantly more prevalent than before labor [46].

In conclusion, while tranexamic acid appears promising for the prevention of PPH after cesareans, the results currently available do not yet justify its widespread use. We propose to perform an adequately powered, multicenter, randomized, placebo-controlled trial to determine what effect, if any, systematic tranexamic acid administration after cesarean delivery has on PPH incidence.

Our hypothesis, patterned after that of the TRAAP trial for vaginal deliveries [22, 39], is that administration of a low dose of tranexamic acid (1 g) in the 3 min after cesarean delivery and after prophylactic uterotonic administration, reduces blood loss and the incidence of both PPH and its complications.

#### Objectives

The aim of this study is therefore to compare the effect of the administration of a low dose of tranexamic acid (1 g) in the 3 min after a cesarean delivery with placebo, both in addition to prophylactic uterotonics, in a multicenter randomized, double-blind, placebo-controlled trial with two parallel groups.

The specific object ives, similar to but modified from those for the TRAAP trial for vaginal deliveries [22, 39], are as follows:

- To assess the effect of tranexamic acid (1 g) on postpartum blood loss after cesarean delivery
  - Primary outcome
    - Incidence of PPH defined by a calculated estimated blood loss > 1000 mL [Calculated estimated blood loss = estimated blood volume × (preoperative hematocrit - postoperative hematocrit)/preoperative hematocrit (where estimated blood volume (mL) = weight (Kg) × 85)] or red blood cell (RBC) transfusion before day 2 (D2) postpartum [5–7]. Preoperative hematocrit will be the most recent hematocrit within 1 week before delivery. Postoperative hematocrit will be measured at D2 (see below).
  - Secondary outcomes
    - Other outcome measures describing postpartum blood loss, including but not limited to calculated blood loss > 500 mL, > 1500 mL and mean total calculated blood loss, mean gravimetrically estimated blood loss by measuring the suction volume and swab weight until discharge from the post-anesthesia care unit (PACU) (i.e., about 2 h after the end of the cesarean) [23] as well as > 500 mL and > 1000 mL; mean gravimetrically estimated blood loss at the end of the cesarean delivery, mean or median number of units of red blood cells transfused, incidence of postpartum iron perfusion, shock, as well as the outcomes previously considered [22, 39]: provider-assessed clinically significant PPH, proportion of women requiring supplementary uterotonic treatment including sulprostone, incidence of postpartum transfusion, incidence of arterial embolization or emergency surgery for PPH, mean peripartum change in hemoglobin and hematocrit as well as hemoglobin drop > 2 g/dL, transfer to intensive care unit (ICU), or death from any cause.
    - To assess the potential adverse effects of tranexamic acid (1 g) after cesarean delivery:
      - Hemodynamic parameters, adverse gastrointestinal events, renal, hepatic, and coagulation function, and any venous or arterial thrombosis in the 3 months after delivery (39).
- To assess women’s satisfaction and psychological status at D2 and D60 [39].

## Methods/design

The methodology and design are very similar to and modified from those of the TRAAP trial for vaginal deliveries [22, 39].

### Recruitment and allocation

The recruitment and allocation, including inclusion and exclusion criteria, are essentially identical to those reported previously [22, 39], modified for a population with cesarean rather than vaginal deliveries.

#### Inclusion criteria

- Age ≥ 18 years
- Cesarean delivery, either before or during labor
- Gestational age ≥ 34 weeks
- Available venous hematocrit value in the week before the cesarean
- Prenatal hemoglobin level in the week before the cesarean > 90 g/l
- Signed informed consent

#### Exclusion criteria

- History of venous (deep vein thrombosis and/or pulmonary embolism) or arterial (angina pectoris, myocardial infarction, or stroke) thrombosis
- History of epilepsy or seizure
- Any known active cardiovascular, renal, or liver disorders
- Autoimmune disease
- Sickle cell disease
- Severe hemorrhagic disease
- Placenta previa
- Abnormally invasive placenta (placenta accreta/increta/percreta)
- Abruptio placentae
- Eclampsia or HELLP (Hemolysis Elevated Liver Low Platelet) syndrome
- In utero fetal death
- Administration of low-molecular-weight heparin or antiplatelet agents in the week before delivery
- Known hypersensitivity to tranexamic acid or concentrated hydrochloric acid
- Failed operative vaginal delivery
- Planned general anesthesia
- Cesarean delivery for the second twin or second/third triplet(s) after vaginal birth of the first twin.
- Poor understanding of the French language

Women will receive individual information in late pregnancy about the trial from obstetricians and midwives during prenatal visits or from anesthetists during the systematic anesthesia visit, or both.

Women may be pre-included (receive information and sign consent forms) for cesareans both before and during labor when the investigator considers that the woman is likely to have a cesarean delivery. The aim of pre-inclusion is to facilitate recruitment in the trial, particularly the recruitment of cesareans during labor, which may be decided upon and performed in emergency situations.

Women will be randomized to receive either 1 g of tranexamic acid (Sanofi Aventis, Paris, France; Marketing authorization number: 3400931157618 [1974, RCP rev 06.08.2018]) or placebo (normal saline, Fresenius Kabi, Sèvres, France; Marketing authorization number: 3400941573941, RCP rev 02.01.2018), at a 1:1 ratio. The Clinical Epidemiology Unit (CEU) of the Bordeaux University Hospital will create and securely hold the randomization list for allocations to the tranexamic acid and placebo groups. As in the previous trial [22, 39], a list of treatment units, containing the type of product (tranexamic acid or placebo) and the corresponding treatment number, will be forwarded to the pharmacy department of the Angers University Hospital, a member of the PPRIGO hospital pharmacists’ consortium (Production Pharmaceutique pour la Recherche Institutionnelle du Grand Ouest). The blinded products will be supplied in numbered, labeled boxes, each containing a 10-mL vial of the study drug (1 g of tranexamic acid or placebo according to the randomization list). All boxes and vials will be identically labeled, and the drug packs will be differentiated only by the treatment number. Accordingly, all participants, including caregivers, will be masked and the women’s safety simultaneously guaranteed*.*

The randomization will be centralized and stratified by center and the timing of the cesarean (before or during labor). Randomization numbers will be attributed through a web platform (Ennov Clinical Software). Once a woman has been included and a randomization number assigned to her, she will retain this number even in case of withdrawal from the study.

### Intervention

As in the previous trial [22, 39] the intervention will be the intravenous administration of a 10-mL blinded vial of the study drug (either 1 g of tranexamic acid or placebo, according to the randomization group), slowly (over 30–60 s), in the 3 min after birth, the routine prophylactic uterotonic administration, and cord clamping, all generally by the anesthesiologist or nurse-anesthetist.

Except for the content of the study drug vial, all aspects of management of cesarean delivery, including the third stage, will be identical in both arms as previously reported [22]:

- Routine prophylactic intravenous injection of 5 or 10 IU oxytocin or 100 micrograms carbetocin (according to the hospital’s policy) at delivery of the anterior shoulder or in the 3 min after birth, as recommended in the national clinical practice guidelines issued by the French College of Gynecologists and Obstetricians [47].

### Other aspects of the cesarean procedure

- Surgeons will be asked to operate with a standardized procedure:
  - ○ Because the Joel-Cohen technique and its variants (Misgav-Ladach technique) have been associated with less blood loss than the use of the Pfannenstiel abdominal incision (and its variants), use of the former techniques will be preferred [48, 49].
  - ○ Because delivery of the placenta with cord traction at cesarean section has been associated with less blood loss than manual removal, cord traction will be preferred in the absence of severe bleeding [50].
- The care providers will decide upon the administration of additional uterotonics and the management of PPH, in a manner consistent with French guidelines [47] and the center’s protocol. In particular, the use of tranexamic acid for the treatment of PPH will be allowed and left to the practitioner’s discretion according to the center’s protocol.
- The method for estimating blood loss will be standardized and include:
  - ○ Recording of the suction blood volume.
  - ○ Weighing of all swabs or material used to measure additional blood loss.
- Finally, because the obstetrician’s level of experience may affect estimated blood loss during or after a cesarean delivery, the obstetrician’s experience, categorized as junior or senior according to years of specialist training, will be recorded [5].

### Outcome measures

#### Primary outcome measure

The trial’s primary outcome will be the incidence of PPH, defined by a calculated estimated blood loss > 1000 mL or RBC transfusion up to D2 [5]. Calculated estimated blood loss will be based on the difference between the preoperative and postoperative packed cell volume (PCV), also known as hematocrit; it will be calculated as follows [5–7]: calculated estimated blood loss = estimated blood volume × (preoperative hematocrit – postoperative hematocrit/preoperative hematocrit (where estimated blood volume (mL) = weight (kg) × 85).

We have chosen this calculation as a quantitative objective measure to estimate blood loss because of the inaccuracy of blood loss estimation for cesareans through other methods, as described above [2, 8–10].

Preoperative hematocrit will be the most recent hematocrit measured within 1 week before delivery. Postoperative hematocrit will be the hematocrit obtained by blood sampling at D2. If hematocrit is not available at D2, then hematocrit at D3 will be considered; if it is also missing, then the postpartum hematocrit closest to D2 in the absence of transfusion will be considered [39].

All women who receive RBC transfusion for PPH between delivery and D2 postpartum are defined to have PPH and meet the criteria for the primary outcome [5]. Calculated blood loss is impossible to determine for these women. Moreover, women only rarely receive an RBC transfusion for a blood loss less than 1000 mL [2, 4, 6, 47, 51]. As a marker of significant maternal morbidity, RBC transfusion is considered equivalent or superior to blood loss greater than 1000 mL [2, 3, 47, 52].

#### Secondary outcomes

- Secondary outcome measures describing postpartum blood loss

#### Clinical

- mean gravimetrically estimated blood loss, by measuring the suction volume and swab weight according to Gai et al. (estimated blood loss = (weight of materials used + materials not used - weight of all materials before surgery)/1.05 + volume included in the suction container) [23, 27, 28], recorded from placental delivery to:
  - ▪ the end of the cesarean delivery, and just before the woman’s transfer to the PACU.
  - ▪ the discharge of the woman from PACU (i.e., about 2 h after the end of the cesarean delivery).
- gravimetrically estimated blood loss > 500 mL
- gravimetrically estimated blood loss > 1000 mL
- incidence of provider-assessed clinically significant PPH, defined by the providers’ response to the question: “Was there a PPH?” [22, 39]
- proportion of women requiring supplementary uterotonic treatment, including sulprostone [22, 39]
- incidence of postpartum transfusion (until discharge) [22, 39]
- mean number of RBC units transfused
- incidence of iron sucrose perfusion (until discharge from hospital)
- incidence of arterial embolization and emergency surgery for PPH [22, 39].
- incidence of hypovolemic shock related to PPH
- incidence of transfer to ICU
- incidence of maternal death from any cause [22, 39].

#### Laboratory

- incidence of calculated blood loss > 500 mL and > 1500 mL
- mean total calculated blood loss
- mean peripartum change in hemoglobin (difference between hemoglobin before delivery and at D2) [22, 39]
- Hemoglobin drop > 2 g/dL (between hemoglobin before delivery and at D2) [22, 39]

For all laboratory indicators, the predelivery reference examination will be the most recent blood count obtained within 1 week before delivery. As previously described [22, 39], all patients included in the trial will provide a blood sample on the second day postpartum (D2) to measure their peripartum hemoglobin and hematocrit and calculate the change in these two indicators. If no blood sample is available from D2, these measurements will be assessed from a D3 blood sample, if available. If no blood sample is available from D2 or D3, they will be assessed from the blood sample closest to D2 in the absence of transfusion.

- The occurrence of potential adverse effects of tranexamic acid, most as previously reported (39), with some modifications:

#### Clinical

- Hemodynamic parameters (heart rate, blood pressure) 15, 30, 45, 60, and 120 min after delivery [22, 39].
- the occurrence of potential mild adverse effects of tranexamic acid in the operating room:
  - ▪ nausea
  - ▪ vomiting
  - ▪ phosphenes
  - ▪ dizziness
- the occurrence of potential severe adverse effects of tranexamic acid during the hospital stay and up to 12 weeks postpartum [22, 39]:
  - ▪ deep vein thrombosis, if the diagnosis is confirmed by Doppler ultrasound [39]
  - ▪ pulmonary embolism, if the diagnosis is confirmed by radiological examination [39].
  - ▪ myocardial infarction [39]
  - ▪ seizure [39]
  - ▪ renal failure requiring dialysis [39]
  - ▪ any other unexpected adverse events [39].

As previously [39], these events will be checked by the medical team during the hospitalization and then at 12 weeks postpartum by a telephone interview of each woman. In the event that the woman cannot be reached, at least 10 calls at different hours over the course of the week will be made to minimize loss to follow-up. In cases of severe adverse effects reported by a woman after discharge, objective data will be collected from medical files, transmitted either by the woman herself or her general practitioner.

#### Laboratory

- mean urea and creatinemia, prothrombin time (PT), active prothrombin time (aPTT), aspartate and alanine transaminase, and total bilirubin at D2 [22, 39].

- Women’s satisfaction and psychological status
  - This will be assessed by a self-administered questionnaire on D2 postpartum and by mail or email at 8 weeks postpartum [22, 39].

### Statistical analysis

As in our previous TRAAP study of vaginal deliveries [22, 39], data analysis and reporting will adhere to the CONSORT guidelines for randomized controlled trials and will be conducted with the trial statistician and researchers blinded to group status. The women’s demographic characteristics and standard risk factors for PPH will be compared between the two groups. The main analysis of the primary and secondary outcomes will be performed in the modified intention-to-treat (ITT) population, defined as women who undergo randomization and have a cesarean delivery (except if they withdraw consent or are deemed ineligible after randomization). We will also analyze two separate per-protocol populations: women from the modified ITT population receiving a uterotonic and then tranexamic acid or placebo in the 3 min after delivery (as prespecified in the protocol) (per-protocol group 1); the other will include women from the modified ITT population receiving a uterotonic and then tranexamic acid or placebo in the 10 min after delivery (per-protocol group 2; for a situation more consistent with routine clinical practice).

The baseline characteristics of the trial participants, the management of the third stage of labor, and protocol adherence will be compared by descriptive statistics. Quantitative variables will be expressed, as appropriate, as means with standard deviations (and compared by Student’s t-test) or as medians with interquartile ranges (compared by the Wilcoxon rank-sum test). Chi-square or Fisher’s exact tests will be used, as appropriate, to compare categorical variables. The effects of tranexamic acid will be expressed as relative risks with 95% confidence intervals for categorical outcomes and as mean differences with 95% confidence intervals for quantitative outcomes. The results will also be expressed as absolute risk differences with 95% confidence intervals for binary outcomes.

Two prespecified subgroup analyses will examine the primary outcome in subgroups of women at high risk of postpartum hemorrhage. The subgroups will include women who undergo a cesarean during labor and those who are at risk for postpartum hemorrhage according to a composite definition (having at least one risk factor with an odds ratio of 3 or greater in the literature [53]: history of any of postpartum hemorrhage, pregnancy-related hypertensive disorder, multiple pregnancy, or cesarean during labor). Moreover, prespecified subgroup analyses will examine the secondary outcomes in the subgroup of women who undergo a cesarean during labor. We will use the Benjamini–Hochberg procedure to adjust for multiple comparisons of secondary outcomes or subgroups.

### Feasibility

Some participating centers have previously collaborated in two French PPH-related multicenter randomized controlled trials assessing the impact of controlled cord traction (TRACOR trial [51]) and/or of tranexamic acid (1 g) after vaginal delivery (TRAAP trial [22]) on the prevention of PPH. Together they have randomized more than 4000 women with vaginal deliveries for each of these trials and shown their capacity to conduct large trials related to PPH prevention. Moreover, all participating centers belong to a national network called Groupe de Recherche en Obstétrique et Gynécologie (GROG) and have recently participated successfully in several RCTs, demonstrating their ability to run large RCTs assessing perinatal outcomes [54–56].

### Sample size

We assumed a 15% incidence of our primary outcome (calculated blood loss > 1000 mL or RBC transfusion before D2) after cesareans in the absence of tranexamic acid, based on the results from a high-quality RCT conducted in the Republic of Ireland with a similar outcome measure (*n* = 2069) [5]. To show a relative reduction of at least 20% in the incidence of postpartum hemorrhage in the tranexamic acid arm — that is, an incidence of 12% or less in this arm, with α = 0.05, 1 − β = 0.80, and a bilateral test, the study will require 2036 women with a cesarean delivery in each group, totaling 4072 participants. A higher number of randomized women must be included given that some will have a scheduled cesarean cancelled, be lost to follow-up, or lack the postpartum blood sample needed to assess the primary outcome (estimated at a maximum of 10%). Accordingly, we plan to include 2262 women in each group, totaling 4524 participants.

### Timetable

The 28 French participating maternity units perform 92,500 deliveries a year. The average cesarean rate is estimated at 25% in these tertiary university centers (20.4% national rate in 2016 [57]). Considering the trial’s exclusion criteria, and in particular the exclusion of cesareans performed before 34 weeks, we estimate that 18% of parturients, i.e., 16,500/year, will be eligible for the trial in the participating centers. An inclusion period of 24 months should make it possible to recruit 4524 women, if we assume a participation rate of at least 15%. This rate appears realistic given the previous patient recruitment obtained in similar trials in some of the participating centers (TRACOR and TRAAP trials) [22, 51].

The trial is expected to last a total of 27 months, including 24 months of inclusions and 3 months of postpartum follow-up.

### Data management

Each investigator will be responsible for ensuring the accurate recording of the data, which will be completed by clinical research technicians (CRTs) throughout the trial, with Clinsight software. The electronic case report file for each woman will contain 5 components, as previously described [39]:

- 1 completed by the CRT from the obstetric file: woman’s characteristics, course of the pregnancy, labor, and delivery.
- 1 completed by the CRT about the postpartum events after leaving the delivery room, and the results of the postpartum blood count.
- 1 questionnaire about women’s satisfaction on D2 postpartum, completed by the women, with responses entered secondarily in the electronic file by the CRT.
- 1 questionnaire about the women’s satisfaction and psychological status at 8 weeks postpartum, sent by the CRT to the women and completed by them, with responses entered secondarily in the electronic file by the CRT.
- 1 questionnaire about the occurrence of thromboembolic and any other unexpected events at 12 weeks in postpartum, completed by the CRT during a telephone interview with the woman, with responses secondarily entered in the electronic file by the CRT.

The Methodology and Data management Centre of the Bordeaux University Hospital will handle the data management and statistics centrally, under the supervision of the Scientific Director of the study (CDT).

Quality control will be conducted according to the standard operating procedures of the sponsor, Bordeaux University Hospital. The research in the investigational centers and management of subjects will comply with the Declaration of Helsinki and Good Clinical Practices. CRTs will conduct regular visits to each investigative center and report to the Data and safety monitoring committee (DSMC). During these onsite inspections and in accordance with Good Clinical Practices and as in the previous TRAAP study [39], the following items will be reviewed:

- Compliance with the research protocol and the procedures defined therein
- Patients’ informed consents, to be verified for all women included
- The source documents to compare with the data reported in the eCRFs for accuracy and consistency of the data and the missing data.
- End-of-trial visit: archiving of research documents.

### Trial steering committee

A trial steering committee (TSC) will be set up, which will be responsible for overall supervision of the trial. It will meet before the trial starts and then at least every 2 months until the trial is completed. The TSC will meet within a month of every Safety Monitoring Committee meeting to consider their recommendations (as modified from [39]).

### Safety consideration

As in the first TRAAP trial [39], an independent Data and Safety Monitoring Committee will also be formed. Its members will meet yearly to examine recruitment figures, baseline data, and will review the result of the interim safety data analysis when half of the planned sample size has been recruited.

Suspected unexpected serious adverse reactions (SUSARs) will be recorded and reported with the ANSM (National Agency for Drug Safety) approved SUSAR form. As in the first TRAAP trial [39], SUSARs include maternal death, surgery (other than cesarean delivery) in the 12 weeks after randomization, transfusion of more than 4 units of blood, admission to an ICU, deep vein thrombosis, pulmonary embolism, myocardial infarction, seizure, renal failure defined by the need for dialysis, or suspected drug reactions. In case of a SUSAR the form will be filled in by the local coordinator and transmitted to the trial coordinating center at the Bordeaux University Hospital within 72 h. Copies of the form will then be sent to the trial statistician and the chair of the Safety Monitoring Committee*.* The Safety Monitoring Committee may ask for unblinding, while the investigators will remain blinded to the treatment allocation. The ANSM, the trial sponsor, and the Chair of the Ethics Committee will also be informed by the Safety Monitoring Committee if considered appropriate, in particular when SUSARS related to the Investigational Medicinal Products are suspected. Finally, if the Safety Monitoring Committee judges it needed, it can recommend to the Scientific Committee that the trial be stopped.

## Discussion

### Potential and implementation of the findings

Reports of increases in PPH incidence in high-resource countries [3, 58, 59] underline the need for prophylaxis that goes beyond the current recommendation for the administration of a uterotonic at the start of the third stage of labor. PPH prevention might be enhanced by a treatment that acts on the coagulation process. Tranexamic acid, as an inexpensive agent that is simple to administer, could easily be added to the routine management of cesarean deliveries worldwide. It is thus a promising candidate for prevention. Nonetheless, the evidence now available does not justify its widespread use in this situation. This large, multicenter, randomized placebo-controlled trial aims to determine with adequate power if the benefits of the routine prophylactic use of tranexamic acid after cesarean delivery significantly outweigh its risks for the safe prevention of PPH.

## Data Availability

Not applicable.

## Abbreviations

ANSM: National Agency for Drug Safety
aPTT: Active prothrombin time
CEU: Clinical epidemiology unit
CI: Confidence interval
CNIL: *Commission Nationale Informatique et Libertés* (French national data protection agency)
CPP: *Comité de Protection des Personnes* (Committee for protection of persons)
CRT: Clinical research technician
D: Day
DSMC: Data and safety monitoring committee.
eCRF: Electronic Case Report Form
g: Gram
GROG: *Groupe de Recherche en Obstétrique et Gynécologie* (Obstetrics and gynecology research group)
HELLP: Hemolysis elevated liver low platelet
ICU: Intensive care unit
IU: International unit,
Kg: Kilogram
L: Liter
mL: Milliliter
PACU: Post anesthesia care unit
PHRC: *Programme Hospitalier de Recherche Clinique* (hospital clinical research program)
PPH: Postpartum hemorrhage
PPRIGO: *Production pharmaceutique pour la recherche institutionnelle du grand ouest* (pharmaceutical production for Western France institutional research)
PT: Prothrombin time
PVC: Packed cell volume
RBC: Red blood cells
RCT: Randomized controlled trial
RR: Risk ratio
SUSARs: Suspected unexpected serious adverse reactions
TRAAP: Tranexamic acid for preventing postpartum hemorrhage following a vaginal delivery (randomized controlled trial)
TRACOR: Traction of the cord (randomized controlled trial)
TSC: Trial steering committee
WOMAN: World maternal antifibrinolytic (randomized controlled trial)
